# SUPPORT Tools for Evidence-informed Policymaking in health 18: Planning monitoring and evaluation of policies

**DOI:** 10.1186/1478-4505-7-S1-S18

**Published:** 2009-12-16

**Authors:** Atle Fretheim, Andrew D Oxman, John N Lavis, Simon Lewin

**Affiliations:** 1 Norwegian Knowledge Centre for the Health Services, P.O. Box 7004, St. Olavs plass, N-0130 Oslo, Norway; Section for International Health, Institute of General Practice and Community Medicine, Faculty of Medicine, University of Oslo, Norway; 2 Norwegian Knowledge Centre for the Health Services, P.O. Box 7004, St. Olavs plass, N-0130 Oslo, Norway; 3 Centre for Health Economics and Policy Analysis, Department of Clinical Epidemiology and Biostatistics, and Department of Political Science, McMaster University, 1200 Main St. West, HSC-2D3, Hamilton, ON, Canada, L8N 3Z5; 4 Norwegian Knowledge Centre for the Health Services, P.O. Box 7004, St. Olavs plass, N-0130 Oslo, Norway; Health Systems Research Unit, Medical Research Council of South Africa

## Abstract

*This article is part of a series written for people responsible for making decisions about health policies and programmes and for those who support these decision makers*.

The term *monitoring *is commonly used to describe the process of systematically collecting data to inform policymakers, managers and other stakeholders whether a new policy or programme is being implemented in accordance with their expectations. Indicators are used for monitoring purposes to judge, for example, if objectives are being achieved, or if allocated funds are being spent appropriately. Sometimes the term *evaluation *is used interchangeably with the term *monitoring*, but the former usually suggests a stronger focus on the achievement of results. When the term *impact evaluation *is used, this usually implies that there is a specific attempt to try to determine whether the observed changes in outcomes can be attributed to a particular policy or programme. In this article, we suggest four questions that can be used to guide the monitoring and evaluation of policy or programme options. These are: 1. Is monitoring necessary? 2. What should be measured? 3. Should an impact evaluation be conducted? 4. How should the impact evaluation be done?

## About STP

*This article is part of a series written for people responsible for making decisions about health policies and programmes and for those who support these policymakers. The series is intended to help such people ensure that their decisions are well-informed by the best available research evidence. The SUPPORT tools and the ways in which they can be used are described in more detail in the Introduction to this series *[1]. *A glossary for the entire series is attached to each article (see Additional File *1*). Links to Spanish, Portuguese, French and Chinese translations of this series can be found on the SUPPORT website *http://www.support-collaboration.org. *Feedback about how to improve the tools in this series is welcome and should be sent to*: STP@nokc.no.

## Scenarios

*Scenario 1: You are a senior civil servant with overall responsibility for several healthcare programmes. You wish to ensure that you have the information necessary to assess how various programmes are performing and the impact they are having*.

*Scenario 2: You work in the Ministry of Health and have been instructed to prepare a memo on various issues that should be taken into consideration when the national vaccination programme is evaluated*.

*Scenario 3: You work in a unit supporting the government in its use of evidence in policymaking. You are preparing a monitoring and evaluation plan for the national tuberculosis control programme*.

## Background

For policymakers (Scenario 1), this article suggests a number of questions that their staff might be asked when planning the monitoring and evaluation of a new policy.

For those who support policymakers (Scenarios 2 and 3), this article suggests a number of questions to consider when planning how to monitor the implementation of policies and programmes, and the evaluation of their impacts.

Policymakers and other stakeholders will often need to know whether a new policy or programme has been implemented in accordance with their expectations. Is the programme rollout progressing as planned? Are the objectives being achieved, and are the allocated funds being spent appropriately? *Monitoring *is the term commonly used to describe the process of systematically collecting data to provide answers to such questions [2]. The term *performance monitoring *is often used when the main focus of an evaluation is comparing "how well a project, program, or policy is being implemented against expected results" [2].

*Indicators *are frequently used as part of the monitoring process. An indicator has been defined as a "quantitative or qualitative factor or variable that provides a simple and reliable means to measure achievement, to reflect the changes connected to an intervention, or to help assess the performance" [2]. An indicator can be a simple count of events, e.g. the number of vaccinations conducted within a set period of time, or a construct based on various data sources, e.g. the proportion of all children being fully immunised before their first birthday.

The term *evaluation *is sometimes used interchangeably with *monitoring*, but the former usually suggests a stronger focus on the achievement of results. These terms are not used consistently and may mean different things to different people. The term *impact evaluation *is frequently used when an attempt is made to evaluate whether observed changes in outcomes (or 'impacts') can be attributed to a particular policy or programme.

## Questions to consider

1. Is monitoring necessary?

2. What should be measured?

3. Should an impact evaluation be conducted?

4. How should the impact evaluation be done?

### 1. Is monitoring necessary?

The importance of monitoring depends on the perceived need among relevant stakeholders to know more about what is happening 'on the ground'.

Determining whether a system for monitoring a policy or programme should be established may depend on several factors, including:

• Whether a monitoring system is already in place that includes the desired indicators, or if a new set of indicators is required

• The likely costs of establishing the system required. For example, could a few new items be added to data collection procedures already in place, or is it necessary to conduct additional large-scale household surveys or to develop a completely new tool?

• Whether the findings are likely to be useful. What actions should be taken if monitoring reveals that things are not going as planned?

Monitoring is not worthwhile if data remain unused. Data are particularly useful if corrective action is undertaken when a gap is identified between expected and actual results. Such findings may result in expectations being reconsidered. This may take the form of assessments, for example, of whether the initial plans were too ambitious, or whether a new policy has failed to work as effectively as expected.

See Table 1 for two illustrative examples of monitoring systems that have been put in place within health systems [3,4]

**Table 1:** Examples of monitoring systems in the healthcare system

**Scaling up provision of antiretroviral therapy (ART) in Malawi **[3]
When Malawian health authorities decided to make ART available to a large proportion of the HIV-positive population, a system was put in place to monitor the implementation of this new policy. The principles of the system are based on the WHO's approach to the monitoring of national tuberculosis programmes. Each patient who starts on ART is given an identity card with a unique identity number, and this is kept at the clinic. The information collected from new patients includes their name, address, age, height, the name of their guardian, and the reason for starting ART. Patients are asked to attend each month to collect their medication. During their visit, their weight is recorded and they are asked about their general health, ambulatory status, work, and any drug side effects. Pill counts are also undertaken and recorded as a way of ensuring drug adherence. In addition, the following standardised monthly outcomes are recorded using the following categories:
• *Alive*: Patient is alive and has collected his/her own 30-day supply of drugs
• *Dead*: Patient has died while on ART
• *Defaulted*: Patient has not been seen at all for a period of 3 months
• *Stopped*: Patient has stopped treatment completely either due to side effects or for other reasons
• *Transfer-out*: Patient has transferred out permanently to another treatment
Data collected as part of the Malawian monitoring system of the ART rollout may be analysed and used in a variety of ways. Comparisons can be made of treatment outcomes for patients who were recruited at different times. If, for example, the rate of switching from first- to second-line regimens increases, or rates of mortality do likewise, an increase in drug resistance to the first-line regimen could be the cause. If the rate of deaths or defaulters declines, this could indicate that the management of the ART treatment programme is improving. If outcomes are particularly poor in certain geographic areas or clinics, action may need to be taken to address this.
**Lung cancer surgery in Denmark **[4]
Danish authorities issued national clinical practice guidelines for the management of lung cancer prompted by poor outcomes for patients who underwent lung cancer surgery. To monitor the implementation of the guidelines, a register of lung cancer patients was established which included specific information about those patients undergoing surgery. Indicators selected by the Danish Lung Cancer Registry include the extent (or 'stage') of cancer in the body, the surgical procedure used, any complications that occurred, and the survival outcome.
Data from the Danish Lung Cancer Registry are used, among other reasons, to monitor whether national recommendations for lung cancer surgery are being followed. Local, regional, and national audits are performed with the purpose of identifying problems or barriers that may impede adherence to the national guidelines. Based on these findings, specific strategies are proposed for quality improvement.

### 2. What should be measured?

**Figure 1:**
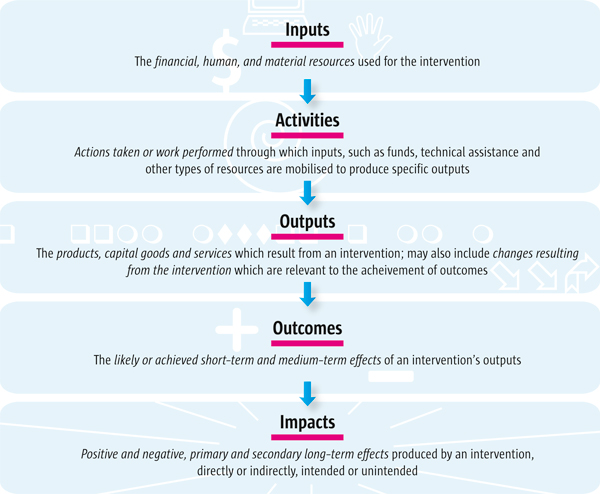
**Results chain-model (definitions adapted from **[2]).

Indicators that focus on various parts of the 'results chain' (i.e. on inputs, activities, outputs, outcomes or impacts - see Figure 1) are typically used to monitor the implementation of a programme or policy option. In some circumstances it may be seen as sufficient to monitor inputs (i.e. the provision of resources such as personnel and equipment). In others it may be important to monitor the activities of the programme or its outcomes (such as the number of children fully immunised).

A number of factors need to be considered when selecting which indicator(s) to use [5,6]:

• *Validity*: the extent to which the indicator accurately measures what it purports to measure

• *Acceptability*: the extent to which the indicator is acceptable to those who are being assessed and those undertaking the assessment

• *Feasibility*: the extent to which valid, reliable and consistent data are available for collection

• *Reliability*: the extent to which there is minimal measurement error, or the extent to which findings are reproducible should they be collected again by another organisation

• *Sensitivity to change*: the extent to which the indicator has the ability to detect changes in the unit of measurement

• *Predictive validity*: the extent to which the indicator has the ability to accurately predict relevant outcomes

Costs related to data collection and the capacity to analyse and feed back data to managers and providers may also limit the choice of indicators. In settings where analytical resources are scarce, it may be preferable to select a simple indicator even if it does not have the best predictive validity, rather than an indicator that requires statistical manipulation.

A trade-off is often apparent between, on one hand, wanting to use desired and optimal indicators and on the other hand, having to use those indicators which are based on existing data. There are good reasons not to select more indicators than are absolutely essential. These reasons include the need to limit the burden of data collection within a health system, avoid the collection of data that are not utilised, and focus on collecting data of higher quality, even if this means collecting less data overall [7].

Routinely collected information from health systems may provide valuable data that can be used as a data source for monitoring purposes. Data can also be collected specifically for the purpose of monitoring, e.g. through surveys or interviews. Consideration should be given to the level of motivation among those expected to collect data. In many instances, health personnel will need to integrate data collection into a busy daily schedule. Therefore if the information being collected has little or no local obvious value to them, their motivation for undertaking such tasks may be low. Similarly, if incentives or penalties are associated with the findings from the monitoring process (e.g. where the payment of providers is linked to performance indicators), the risk of data manipulation or system gaming should be considered.

### 3. Should an impact evaluation be conducted?

**Figure 2:**
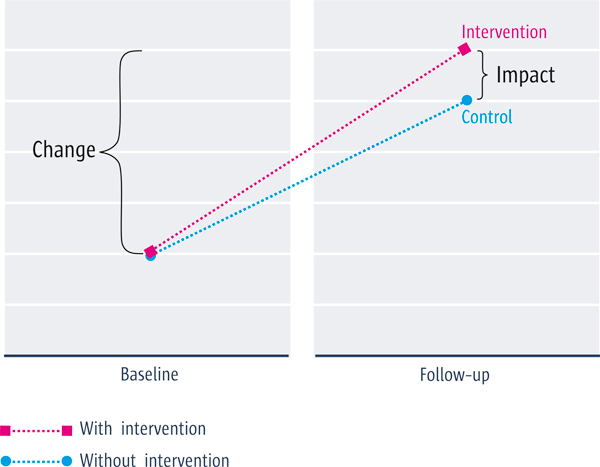
Comparing change in performance in two areas: one with an intervention and one without*. * The Figure illustrates that attributing the change from 'Baseline' to 'Follow-up' in response to an intervention is likely to be misleading. This is because, in this instance, there is also an improvement in the 'Control'. Even with regard to the Control, it is uncertain whether the difference between the 'Intervention' and 'Control' (i.e. the 'Impact') can, in fact, be attributed to the programme or intervention. There may be other differences between the 'Intervention' and 'Control' settings that might have led to the observed difference in the indicator measured

One of the limitations of monitoring activities, as described above, is the fact that such activities do not necessarily indicate whether a policy or programme has had an impact on the indicators that have been measured. This is because indicators used for monitoring will almost always be influenced by factors other than those related to particular interventions. This makes it extremely difficult to determine which factors caused the observed changes. If monitoring reveals that performance is improving, this does not necessarily mean that the intervention is the (only) causal factor. It is conceivable that the indicators would have improved anyway even in the absence of the intervention (see Figure 2).

The establishment of a causal relationship between a programme or policy and changes in outcomes is at the core of what impact evaluation is about. What would have happened to those receiving an intervention if they had not in fact received it, is *the *central impact evaluation question, according to the World Bank [8].

There may be strong reasons to expect positive results based on solid documentation from, for example, previous evaluations. However, very often such evidence is lacking. Or the evidence available may not be applicable to the current setting. Thus, there is a real risk that a new programme may be ineffective or, even worse, cause more harm than good. This issue is important for policymakers to clarify when implementing new programmes. It is also important because of the benefit that such knowledge could bring to future health policymaking both in the programme setting and other jurisdictions.

Conducting impact evaluations can be costly. Whether such studies represent good value for money can be ascertained by comparing the consequences of undertaking an evaluation with the consequences of not undertaking an evaluation. For example, is it likely that a programme would be stopped or modified if the results proved to be negative? If the answer is 'no', the value of undertaking an impact evaluation is clearly limited.

An impact evaluation is generally more likely to represent value for money when results can be obtained as the intervention is being rolled out. In such circumstances there is an opportunity to improve or stop the rollout based on the results of an impact evaluation conducted in the early stages of implementation. This would provide value for money in two instances: firstly, when a pilot study is not possible and, secondly, when it would be possible and practical to modify or stop the rollout (if needed) based on the results.

The Mexican government's health insurance scheme, Seguro Popular, is an example of an impact evaluation embedded in a programme rollout [9-11]. Implemented in 2001, the scheme was established in order to extend health insurance coverage to the almost 50 million Mexicans not yet covered by existing programmes. Taking advantage of the timetable of the progressive rollout, the government set up an evaluation comparing the outcomes for those communities receiving the scheme with those still waiting for it. In addition to evaluating whether the reform achieved the outcomes intended and did not have unintended adverse effects, the evaluation also provides for shared learning.

An impact evaluation may also be useful after a programme has been fully implemented, e.g. when there is uncertainty about continuing a programme. For example, the conditional cash transfer scheme, Progresa (later known as Oportunidades), which was introduced in the mid-1990s provided cash "on the condition that families fulfil particular elements of co-responsibility, such as sending children to school rather than work, providing them with a specially formulated nutritional supplement, and attending a clinic to receive a specified package of interventions for health promotion and disease prevention." [12]. For evaluation purposes, 506 communities were randomly assigned to either enter the programme immediately or 2 years later [13]. The findings from this impact evaluation directly informed policy decisions in Mexico, persuading the government "not only to continue with the programme, but also to expand it" [12].

### 4. How should the impact evaluation be done?

Attributing an observed change to a programme or policy requires a comparison between the individuals or groups exposed to it, and others who are not. It is also important that the compared groups are as similar as possible in order to rule out influences other than the programme itself. This can effectively be done by randomly allocating individuals or groups of people (e.g. within geographic areas) to either receive the programme or not to receive it, in what is called a *randomised trial*. Usually such trials are conducted as pilot projects before a programme is introduced at a national level. But they can also be undertaken in parallel with full scale implementation, as illustrated by the Mexican examples given above.

Randomised trials may, however, not always be feasible. Alternative approaches include the comparison of changes before and after programme implementation, with observed changes during the same time period in areas where the programme was not implemented (e.g. in neighbouring districts or countries). This is called a *controlled before-after evaluation*. Alternatively, an *interrupted time-series *may be used in which data are collected from multiple time points before, during, and after programme implementation.

Simply comparing the value of an indicator before and after programme implementation is not generally recommended since the risk of misleading findings is high - observed changes, e.g. HIV-incidence may be caused by known and unknown factors other than those related to the programme itself (see Figure 2) [14,15]. An overview of a number of evaluation designs is provided in Additional File 2. The weaknesses and strengths of each method described in Additional file 2 are outlined in Additional file 3.

Impact evaluations should be planned well ahead of programme implementation in conjunction with relevant stakeholders, including policymakers. After a programme has been rolled out widely it is usually too late to carry out baseline measurements or to establish appropriate comparison groups. For example, using random assignments to decide whether communities will be included in a programme or not, cannot be done after the programme has been implemented nationally. Impact evaluations that are built into a programme from the start are thus more likely to yield valid findings than those evaluations conducted as an afterthought. Furthermore, if impact evaluations are seen as an integrated part of programme implementation, policymakers and others may be more committed to taking the findings into account.

The number of individuals or communities required for an impact evaluation should also be estimated at an early stage. This will ensure that there is sample size large enough for meaningful conclusions to be drawn from the evaluation findings.

In healthcare, as in most other areas, programmes need to be both effective *and *cost-effective. To assess the economic aspects of a programme, resource use and costs must be estimated, preferably based on data collected from real-life implementation [16]. Decisions on what economic data to collect should therefore also be made at an early stage, before the evaluation starts.

Impact evaluations are likely to be most informative if a process evaluation is included. A process evaluation may examine whether the programme or policy option was delivered as intended. It may also investigate the processes of implementation and change, explore responses to the programme, and explore reasons for the findings of the evaluation [17].

See Table 2 for examples of impact evaluations.

**Table 2:** Examples of impact evaluations

**Home-based antiretroviral therapy (ART) in Uganda **[20-22]
Shortages of clinical staff and difficulties with accessing care due to transportation costs are major obstacles to scaling up the delivery of ART in developing countries. One proposed solution is home-based HIV care, where drug delivery, the monitoring of health status, and the support of patients is carried out at the home of the patient by non-clinically qualified staff. It is highly uncertain, however, whether this strategy is able to provide care of sufficient quality, including timely referrals for medical care, or whether such a system is cost-effective. Therefore, before implementing home-based care programmes widely it is important that they are evaluated for their (cost-) effectiveness.
To ensure a fair comparison between home-based and facility-based ART, researchers in Uganda conducted a randomised trial. The study area was divided into 44 distinct geographical sub-areas. In some of these, home care was implemented, while in others a conventional facility-based system continued to be used. The selection and allocation of areas to receive, and not to receive, the home-based care system, was randomly determined. This reduced the likelihood of important differences between the comparisons groups which might otherwise have influenced the study if, for example, the districts themselves had decided whether to implement home-based care, or if decisions had been based on an existing preparedness to implement home-based care. The random allocation system used was also the fairest way of deciding where to start home-based care since each district had an equal chance of being chosen.
The researchers found that the home-based care model using trained layworkers was as effective as nurse- and doctor-led clinic-based care.
**Mandatory use of thiazides for hypertension in Norway **[23]
As a cost-containment measure, policymakers in Norway decided that thiazides would be prescribed as anti-hypertensive drugs instead of more costly alternatives, in those instances where drug expenses were to be reimbursed. The policy was implemented nationally a few months after the decision was made. Because critics continued to argue that the new policy was unlikely to lead to the expected results, The Ministry of Health sponsored a study to assess the impact of the policy they had implemented.
The mandatory prescription of thiazides for treating hypertension was implemented across Norway with an urgency that made a planned, rigorous impact evaluation impossible to conduct. However, by accessing the electronic medical records of 61 clinics at a later stage, researchers extracted prescription data ranging from one year before to one year after the new policy was introduced. They analysed the data using an interrupted time-series. Monthly rates of thiazide prescribing and other outcomes of interest were analysed over time to see if any significant changes could be attributed to the implemented policy. Analysis indicated that there was a sharp increase in the use of thiazides (from 10 to 25% over a pre-specified three month transition period), following which the use of thiazides levelled off.

Budget, time or data constraints may act as disincentives to ensuring rigorous implementation of an evaluation. Such constraints can affect the reliability of impact evaluations in a number of ways:

• By compromising the overall validity of the results, for example, due to insufficient planning or follow-up, or through a paucity of baseline data, a reliance on inadequate data sources, or the selection of inappropriate comparison groups

• Through the use of inadequate samples, e.g. due to the selection of samples that are convenient to sample but may not be representative, as a result of sample sizes being too small, or by a lack of sufficient attention to contextual factors

Such constraints can be addressed by starting the planning process early or finding ways to reduce the costs of data collection. It is important to ensure, however, that neither the possible threats to the validity of the results, nor the limitations of the sample, are such that the results of the evaluation will be unable to provide reliable information. Before conducting an evaluation, an assessment should therefore be made as to whether an adequate evaluation is possible. If it is not, an assessment needs to be undertaken as to whether a programme should be implemented without prior evaluation, in the face of uncertainty about its potential impacts [18].

Impact evaluations are not worthwhile if the findings are not used. Results should be used to inform decisions about whether to continue, change or stop existing programmes. Clearly, other interests will also need to be taken into consideration. For instance, decision makers may elect not to emphasise particular findings from certain evaluations when such findings conflict with other interests that are perceived as more important [19]. However, it is important to avoid the suppression of findings from impact evaluations, e.g. for political reasons. Failing to use evaluation findings contradicts one of the main objectives of conducting such evaluations: to learn from experience and share the knowledge that has been generated. Using independent parties to conduct impact evaluations may decrease the risk of having the findings manipulated or held back from the public.

## Conclusion

A number of aspects related to monitoring and evaluation have been described in this article. At present, many programme monitoring and evaluation efforts are commonly done using methods that do not yield valid assessments of the implementation of a policy or programme or valid estimates of effects. Sometimes such evaluations are not done at all. By taking the issues described in this article into consideration, policymakers and those who support them should be able to develop plans that will generate new and directly useful knowledge.

## Resources

### Useful documents and further reading

Segone M (ed). Bridging the gap: The role of monitoring and evaluation in evidence-based policy making. UNICEF, the World Bank and the International Development Evaluation Association. http://www.unicef.org/ceecis/evidence_based_policy_making.pdf

MacKay K. How to Build M&E Systems to Support Better Government. 2007. Washington DC, The World Bank. http://www.worldbank.org/ieg/ecd/docs/How_to_build_ME_gov.pdf

Monitoring and Evaluation (M&E): Some Tools, Methods and Approaches. 2004. Washington DC. The World Bank. http://lnweb90.worldbank.org/oed/oeddoclib.nsf/24cc3bb1f94ae11c85256808006a0046/a5efbb5d776b67d285256b1e0079c9a3/$FILE/MandE_tools_methods_approaches.pdf

Framework for Managing Programme Performance Information. 2007. National Treasury of South Africa. http://www.treasury.gov.za/publications/guidelines/FMPI.pdf

Barber S. Health system strengthening interventions: Making the case for impact evaluation. 2007. Geneva, Alliance for Health Policy and Systems Research. http://www.who.int/alliance-hpsr/resources/Alliance%20%20HPSR%20-%20Briefing%20Note%202.pdf

Savedoff WD, Levine R, Birdsall N. When will we ever learn? Improving lives through impact evaluation. Report of the Evaluation Gap Working Group. 2006. Washington DC, Center for Global Development. http://www.cgdev.org/content/publications/detail/7973/

Grimshaw J, Campbell M, Eccles M and Steen N. Experimental and quasi-experimental designs for evaluating guideline implementation strategies. Family Practice 2000; 17: S11-S18. http://fampra.oxfordjournals.org/cgi/reprint/17/suppl_1/S11

### Links to websites

- Independent Evaluation Group (IEG) at the World Bank: http://www.worldbank.org/ieg - IEG is an independent unit within the World Bank. IEG assesses what is effective or not effective with regard to policy options, how a borrower plans to run and maintain a project, and the lasting contribution of the Bank to a country's overall development.

- International Initiative for Impact Evaluation (3ie): http://www.3ieimpact.org - 3ie seeks to improve the lives of poor people in low- and middle-income countries by providing and summarising evidence related to what policy options work, as well as when and why, and the costs involved.

- Health Metrics Network: http://www.who.int/healthmetrics/en - The Health Metrics Network (HMN) has the strategic goal of increasing the availability and use of timely and accurate health information. To achieve this, HMN identifies strategies for Health Information System (HIS) development and strengthening, supports countries in implementing HIS reform, and increases knowledge about global public goods through research, technical innovation, and sharing lessons learned.

- NorthStar: http://www.rebeqi.org/?pageID=34&ItemID=35 - NorthStar is a tool for planning, conducting and evaluating quality improvement programmes.

## Competing interests

The authors declare that they have no competing interests.

## Authors' contributions

AF prepared the first draft of this article. ADO, JNL and SL contributed to drafting and revising it.

## Supplementary Material

Additional file 1 Glossary Click here for file

Additional file 2 Click here for file

Additional file 3 Click here for file
