# Supplementary material for: SUPPORT Tools for Evidence-informed Policymaking in health 18: Planning monitoring and evaluation of policies
Source: Health Res Policy Syst. 2009 Dec 16;7(Suppl 1):S18. doi: 10.1186/1478-4505-7-S1-S18 (PMC3271828; doi:10.1186/1478-4505-7-S1-S18)
Supplement: Additional file 2 [file 1478-4505-7-S1-S18-S2.doc]

**Additional File 2. Evaluation designs (adapted from the Cochrane Handbook for Systematic Reviews of Interventions*)**

| **Randomised controlled trial** | - An experimental study in which individuals are randomly allocated to receive different interventions (e.g. using the toss of a coin or a list of random numbers generated by a computer) |
| --- | --- |
| **Cluster randomised trial** | - An experimental study in which groups of people (e.g. school classes or hospitals) are randomly allocated to receive different interventions |
| **Non-randomised controlled trial** | - An experimental study in which people are allocated to different interventions using methods that are not random (e.g. patients admitted during Week 1 receive intervention A, those admitted in Week 2 receive intervention B, those in Week 3 receive intervention A again, and so on) |
| **Controlled before-and-after study** | - A study in which observations are made before and after the implementation of an intervention, both in a group that receives the intervention and in a control group that does not. Data collection should usually be done concurrently in the two groups |
| **Interrupted-time-series study** | - A study using observations at multiple time points before and after an intervention. Measurements are *interrupted* by the intervention. The design attempts to detect whether an intervention has had an effect significantly greater than any underlying trend over time |
| **Historically controlled study** | - A study comparing a group of participants receiving an intervention with a similar group from the past who did not |
| **Cohort study** | - A study in which a defined group of people (the cohort) is followed over time, to examine associations between different interventions received and subsequent outcomes. A *prospective* cohort study recruits participants before any intervention and follows them into the future. A *retrospective* cohort study identifies subjects from past records, describing the interventions received and follows them from the time of those records |
| **Case-control study** | - A study comparing people with a specific outcome of interest (*cases*) with people from the same source population but without that outcome (*controls*), to examine the association between the outcome and prior exposure (e.g. receiving an intervention). This design is particularly useful when the outcome is rare |
| **Cross-sectional study** | - A study collecting information on past or present interventions and current health outcomes for a group of people at a particular point in time. This kind of study examines associations between the outcomes and exposure to interventions |
| **Qualitative study** | - A study conducted in a natural setting which is usually designed to interpret or make sense of phenomena in terms of the meanings people bring to them. Typically in such a study, narrative data are collected from individuals or groups of ‘informants’ or from documents. These are then interpreted by the researcher(s) |

* Cochrane Collaboration. Cochrane Handbook for Systematic Reviews of Interventions. Chichester: The Cochrane Collaboration and John Wiley & Sons Ltd.; 2008
