# Supplementary material for: SUPPORT Tools for Evidence-informed Policymaking in health 18: Planning monitoring and evaluation of policies
Source: Health Res Policy Syst. 2009 Dec 16;7(Suppl 1):S18. doi: 10.1186/1478-4505-7-S1-S18 (PMC3271828; doi:10.1186/1478-4505-7-S1-S18)
Supplement: Additional file 3 [file 1478-4505-7-S1-S18-S3.doc]

**Additional File 3. Selected strengths and weaknesses of evaluation designs**

|  | **Strengths** | **Weaknesses** |
| --- | --- | --- |
| **Randomised controlled trial** | - Widely considered to be the strongest design for establishing cause-effect relationships, which is the key focus of impact evaluation | - May be time consuming and represent logistical challenges - The results are not necessarily transferable to settings outside the study setting |
| **Cluster randomised trial** | - Same strengths as for ordinary randomised trials. In addition, the risk of ‘contamination’ is reduced e.g. that intervention A may be received by, or affect, individuals allocated to receive intervention B only. For example, if nurses are allocated randomly to implement a new routine, other nurses may be influenced by these changes and may start undertaking the same activities. It may therefore be better to randomise wards, and all of the staff within them, rather than individual nurses | - Baseline differences may be a problem as the number of units (or *clusters*) that are randomised would usually be lower than in a trial where individuals are randomised. May be time consuming and logistically challenging, but less so than an ordinary randomised trial |
| **Non-randomised controlled trial** | - May be easier and more practical to conduct than a randomised controlled trial | - When allocation is not done using random methods, selection biases may occur, e.g. because patients and health workers adjust their behaviour to the allocation procedure if they prefer one intervention to another |
| **Controlled before-and-after study** | - May be the only practical option, e.g. for large-scale interventions where randomisation is not feasible for practical or political reasons | - Known or unknown differences between the groups that are compared may exert more influence on the findings than the fact that they received different interventions. Consequently, drawing conclusions about cause-effect relationships may be risky - Requires the availability of baseline data |
| **Interrupted-time-series study** | - May be feasible and relatively easy to conduct if the necessary data are made available. No control group required | - The effect size is always difficult to estimate in such analyses because influences other than the intervention under investigation may impact on the observed changes |
| **Historically controlled study** | - May be quickly and easily done if the necessary data are available | - Known or unknown differences between the groups that are compared may exert more influence on the findings than the fact that they received different interventions. Consequently, drawing conclusions about cause-effect relationships is risky |
| **Cohort study** | - Often large studies with a high degree of external validity (i.e. the findings can be generalised). Often conducted over several years, which makes it possible to detect the long-term effects of an intervention | - Cohort studies are typically lengthy and costly, mainly due to the need for following up the (usually) high number of participants - Known or unknown differences between the groups that are compared may exert more influence on the findings than the fact that they were exposed to different interventions. Consequently, drawing conclusions about cause-effect relationships is risky |
| **Case-control study** | - More quickly and easily done than cohort studies | - The retrospective nature of such studies entails collecting information about events that occurred earlier. Such time delays may be a source of error - Known or unknown differences between the groups that are compared may exert more influence on the findings than the fact that they received different interventions. Consequently, drawing conclusions about cause-effect relationships is risky |
| **Cross-sectional study** | - Requires no follow-up time and can therefore be conducted quickly and often at a low cost | - Known or unknown differences between the groups that are compared may exert more influence on the findings than the fact that they received different interventions. Consequently, drawing conclusions about cause-effect relationships is risky |
| **Qualitative study** | - Allows for the collection of more in-depth information than other quantitative designs. Enables an understanding of how interventions and programmes are (or are not) working | - Does not generate data that can be used to estimate the effect of an intervention that are beyond the perception of those who are interviewed or surveyed |
